# Attenuation Correction Using Deep Learning and Integrated UTE/Multi-Echo Dixon Sequence: Evaluation in Amyloid and Tau PET Imaging

**DOI:** 10.1007/s00259-020-05061-w

**Published:** 2020-10-27

**Authors:** Kuang Gong, Paul Kyu Han, Keith A. Johnson, Georges El Fakhri, Chao Ma, Quanzheng Li

**Affiliations:** 1 Gordon Center for Medical Imaging, Massachusetts General Hospital, Boston, MA 02114; 2 Department of Neurology, Massachusetts General Hospital, Boston, MA 02114; 3 Center for Alzheimer Research and Treatment, Department of Neurology, Brigham and Women’s Hospital, Boston, MA 02115

**Keywords:** PET/MR, Attenuation correction, Deep learning, Convolutional neural network, UTE/Multi-Echo Dixon

## Abstract

**Purpose:**

PET measures of amyloid and tau pathologies are powerful biomarkers for the diagnosis and monitoring of Alzheimer’s disease (AD). Because cortical regions are close to bone, quantitation accuracy of amyloid and tau PET imaging can be significantly influenced by errors of attenuation correction (AC). This work presents an MR-based AC method that combines deep learning with a novel ultrashort time-to-echo (UTE)/Multi-Echo Dixon (mUTE) sequence for amyloid and tau imaging.

**Methods:**

Thirty-five subjects that underwent both 11C-PiB and 18F-MK6240 scans were included in this study. The proposed method was compared with Dixon-based atlas method as well as other magnetization-prepared rapid acquisition with gradient echo (MPRAGE)- or Dixon- based deep learning methods. The Dice coefficient and validation loss of the generated pseudo-CT images were used for comparison. PET error images regarding standardized uptake value ratio (SUVR) were quantified through regional and surface analysis to evaluate the final AC accuracy.

**Results:**

The Dice coefficients of the deep learning methods based on MPRAGE, Dixon and mUTE images were 0.84 (0.91), 0.84 (0.92), and 0.87 (0.94) for the whole-brain (above-eye) bone regions, respectively, higher than the atlas method of 0.52 (0.64). The regional SUVR error for the atlas method was around 6%, higher than the regional SUV error. The regional SUV and SUVR errors for all deep learning methods were below 2%, with mUTE-based deep learning method performing the best. As for the surface analysis, the atlas method showed the largest error (> 10%) near vertices inside superior frontal, lateral occipital, superior parietal and inferior temporal cortices. The mUTE-based deep learning method resulted in the least number of regions with error higher than 1%, with the largest error (> 5%) showing up near the inferior temporal and medial orbitofrontal cortices.

**Conclusion:**

Deep learning with mUTE can generate accurate AC for amyloid and tau imaging in PET/MR.

## INTRODUCTION

Amyloid deposits and tau neurofibrillary tangles (NFTs), the two neuropathological hallmarks of Alzheimer’s disease (AD), accumulate decades before neurodegeneration and symptomatic onset and are essential signs for early AD diagnosis. Positron emission tomography (PET) is a powerful imaging tool that has been used to identify and visualize amyloid deposits and progression for over a decade [1, 2]. Recently, high-affinity radiolabels have also been successfully developed for PET imaging of tau NFTs [3, 4]. For both amyloid and tau PET imaging, accurate quantification of the regional cortical uptake is critical for the diagnosis and progression monitoring of AD.

Simultaneous PET/magnetic resonance (MR) have been readily used for neurological applications due to its functional and metabolic imaging capability from PET and MR, excellent soft tissue contrast from MR, as well as benefits in partial-volume and motion corrections. As MR signals arising from protons are not directly related to photon attenuation coefficients, no simple transforms can perform PET attenuation correction (AC) based on MR images. Because the cortical regions are close to the bone, quantitation accuracy of amyloid and tau imaging can be significantly compromised by AC errors in PET/MR. In addition, standardized uptake value ratio (SUVR), a metric widely used for assessment in amyloid and tau PET imaging, can be easily affected by AC errors since the cerebellum cortex (i.e., reference region widely used to calculate SUVR) is close to complicated bone structures (e.g., mastoid process) of the skull [5]. To leverage the quantitative merits of PET/MR for amyloid and tau imaging, accurate AC is indispensable.

Various methods have been proposed for AC in PET/MR [6, 7]. One category of methods segments MR images (e.g., T1-weighted, Dixon, or ultrashort time-to-echo [UTE]/ zero time-to-echo [ZTE] images) into different tissue types (i.e., water, fat and bone) and assigns attenuation coefficient to each tissue type to produce the attenuation map [8–13]. Another category of methods produces attenuation maps by non-rigidly registering MR images to atlas generated from populational computed tomography (CT) and MR image pairs [14–17]. Machine learning and joint estimation methods have also been explored [18–21]. Recently, a UTE/multi-echo Dixon (mUTE) sequence-based method has been proposed to take full advantage of MR physics for AC [22]. The mUTE sequence integrates UTE for imaging short-T2 components (i.e., bone) and multi-echo Dixon for robust water/fat separation into a single acquisition, which enables a physical compartmental model to estimate continuous distributions of attenuation coefficients. However, deficiency exists in the abovementioned MR AC methods. For the segmentation-based method, segmentation errors were observed at boundaries between soft tissue, bone and air, especially near nasal and mastoid-process regions [23, 24]. The atlas-based method cannot account for pathological regions which are not part of the atlas, and is vulnerable to registration errors and time-consuming [10, 25]. Accuracy of the joint-estimation method depends on time-of-flight (TOF) resolution and its computational complexity is another concern. For the mUTE sequence, parameter estimation based on the physical compartmental model is complicated and nontrivial.

Recently, deep learning has shown promising results for synthetic data generation. Various studies based on convolutional neural networks (CNNs) have been proposed for pseudo-CT generation, and showed better results compared to conventional methods for the brain [26–33], pelvis [34, 35], and whole-body [36] regions. Specifically, our group has developed a novel Group-Unet structure [28] which can efficiently handle scenarios with multiple network-input channels [28] and utilize network parameters more efficiently, compared to the popular U-net structure [37]. However, the deep learning methods were mostly assessed using ^18^F-FDG datasets. It is unclear how could these methods work for amyloid and tau PET imaging, which has higher accuracy demand for PET AC along with the need of comprehensive regional and pixelwise analysis. In addition, existing deep learning methods used Dixon/T1-weighted MR images as the network input. It is unclear whether using more AC-specific MR images as the network input can generate improved results.

In this work, we propose a new deep learning-based AC method, CNN-mUTE, for amyloid and tau imaging by leveraging physics information from the mUTE sequence and the efficiency of Group-Unet in handling multiple input channels of mUTE MR images. The performance of the proposed method was evaluated using in vivo imaging data acquired from thirty-five subjects that underwent both ^11^C-PiB and ^18^F-MK6240 scans. We compared the performance of the proposed method with Dixon-based atlas method available in clinical PET/MR scanners, as well as deep learning-based AC methods that use T1-weighted image and Dixon as network input. Freesurfer [38] was used to derive accurate personalized brain masks to evaluate regional errors related to amyloid and tau imaging. Surface analysis was additionally performed to understand errors for the whole cortical regions.

## MATERIALS AND METHODS

### Participates

Thirty-five subjects (three patients with mild-cognitive impairment and thirty-two cognitively healthy volunteers, 19 males and 16 females, 68.6±11.6 years old [range 47–86]) recruited for Harvard Aging Brain Study (HABS) were scanned under a study protocol that was approved by Massachusetts General Hospital (MGH) Institutional Review Board (IRB). Written informed consent was obtained from all subjects before participation in the study.

### MR acquisition

MR acquisitions were performed on a 3T MR scanner (MAGNETOM Trio, Siemens Healthcare, Erlangen, Germany) using body coil for transmission and 12-channel head coil for reception. T1-weighted anatomical images were acquired using magnetization-prepared rapid acquisition with gradient echo (MPRAGE) sequence. 3D mUTE sequence (Supplementary Fig. 1) [22] was used to acquire Dixon and mUTE images, with the following imaging parameters: image size = 128×128×128, voxel size = 1.875×1.875×1.875 mm^3^, hard radiofrequency (RF) pulse with flip angle = 15° and pulse duration = 100 μs, repetition time (TR) = 8.0 ms, maximum readout gradient amplitude = 19.57 mT/m, maximum readout gradient slew rate = 48.9 mT/m/ms, and acquisition time = 52 s. The final reconstruction from the mUTE sequence resulted in seven images: one UTE image and six multi-echo Dixon images with corresponding echo times (TEs) of 0.07, 2.1, 2.3, 3.6, 3.7, 5.0, and 5.2 ms, respectively.

### PET acquisition

Two separate PET/CT examinations were performed for each subject on a whole-body PET/CT scanner (Discovery MI, GE Healthcare, Milwaukee, Wisconsin, USA): one is amyloid PET imaging by the administration of ^11^C-PiB and the other is tau PET imaging based on ^18^F-MK-6240. For amyloid imaging, the imaging protocol consisted of 555 MBq bolus injection of ^11^C-PiB followed by a dynamic scan over 70 min. PET Data from 55–60 min post injection were used for evaluation. For tau imaging, the imaging protocol consisted of 185 MBq bolus injection of ^18^F-MK-6240 followed by a dynamic scan over 120 min. Only data from 85–90 min post injection were evaluated. For both amyloid and tau imaging, PET images were reconstructed using the ordered subset expectation maximization (OSEM) algorithm with point spread function (PSF) modeling with two iterations and sixteen subsets. The voxel size was 1.17×1.17×2.80 mm^3^ and the image size was 256×256×89. The same CT imaging protocol was used for both amyloid and tau imaging, with tube peak voltage = 120 kVp, tube current time product 30 = mAs, in-plane resolution = 0.56×0.56 mm^2^, and slice-thickness = 1 mm.

### Neural network details

For the Unet structure [37], the feature size after the first convolutional module needs to be enlarged to fully utilize the input information from multi-contrast MR images or neighboring slices, which increases the total number of parameters dramatically. In this work, Group-Unet structure was employed to efficiently handle scenarios with multiple network-input channels [28] and to utilize network parameters more efficiently, based on the assumption that when the network goes deeper, the spatial information becomes discrete and the traditional convolution module can be replaced by the group convolution module [39]. Details of the Group-Unet structure are shown in Supplementary Fig. 2.

To construct the training pairs, MR images were registered to CT images through rigid transformation using the ANTs software [40]. Random rotation and permutation were performed on the training pairs to avoid over-fitting. When random rotation was applied, interpretation was performed with the voxel size and image size fixed. Though image resolution will be compromised a little due to the interpretation operation, this will not be a problem for attenuation map generation as the attenuation map does not have complicated structures as nature images. The Group-Unet employed in this work was based on 2D convolutions, instead of 3D convolutions, to reduce the GPU memory usage and training parameters, and hence was a 2D network. As PET AC is based on 3D CT images, the network outputs were further combined to construct 3D pseudo-CT images. To reduce the axial aliasing artefacts, four neighboring slices were supplied as the additional network input [41]. With Dixon and MPRAGE images as the network input, the input channel was 5 (neighboring slices); with mUTE images as the network input, the input channel was 5 (neighboring slices) × 7 (number of images). As a result, two network structures were needed due to the varying of network input channels. The only difference between the two networks was the number of input channels (5 vs. 35). Other settings of the network, e.g. number of features after each convolution, were the same. The training parameters were 6.02 million and 6.03 million for the network with 5 input channels and the network with 35 input channels, respectively.

The training objective function was based on the L1-norm loss calculated between the pseudo and the ground-truth CT images. The network was implemented in TensorFlow 1.14 with the Adam optimizer. The learning rate and the decay rates of the default settings in TensorFlow were used. Five-fold cross-validation was utilized to make full use of all the data. The batch size was set to 30, and 600 epochs were used as the training cost function becomes steady after 600 epochs. The training time running 600 epochs was 6.7 hours based on the Nvidia GTX 1080 Ti GPU.

### Pseudo-CT image analysis

To evaluate the quality of generated pseudo-CT images, we first calculated the relative validation loss of the CT images as

(1) $$\mathrm{Relative}\;\mathrm{validation}\;\mathrm{loss}=\frac{\left|CT_{\mathrm{generated}\;}-CT_{\mathrm{truth}\;}\right|}{\left|CT_{\mathrm{truth}\;}\right|},$$

where *CT_truth_* is the ground-truth CT image and *CT_generated_* is the generated pseudo-CT image. As bone regions are close to the cortex, they were additionally quantified using the Dice coefficient. Regions with attenuation coefficient higher than 0.1083 cm^−1^ (200 HU unit) were classified as the bone area.

### PET image analysis

To enable better registration between PET and MR images, the PET image of interest was first registered to the first 8-min frame of the dynamic scan, through which it was registered to the MPRAGE image based on rigid registration. Freesurfer was used for cortical parcellation based on the MPRAGE image to get the region of interests (ROIs) for amyloid and tau quantification. Based on the Braak staging [42], the cortical regions crucial to amyloid burden calculation [43, 44] —superior frontal, rostral anterior cingulate, posterior cingulate, precuneus, inferior parietal, supramarginal, medial orbitofrontal, middle temporal and superior temporal—were used as ROIs for amyloid quantification. As for the quantification of tau imaging, the early Braak stage-related cortices—hippocampus, entorhinal, parahippocampal, inferior temporal, fusiform, posterior cingulate, lingual and insula—were employed as the evaluation ROIs. The ROIs for the amyloid imaging quantification were mostly near the upper regions of the brain, and the ROIs chosen for the tau imaging quantification were concentrated near the middle/inferior temporal regions. By evaluating the AC quality of both amyloid and tau imaging, the performance of different AC methods was examined for most of the cortex regions.

Compared to amyloid deposits which are more heterogeneously and randomly distributed, the pathway of tau NFTs is more precise. Resolving the topographic patterns of tau retention in PET can enable better disease monitoring, which makes surface analysis a necessary addition to regional analysis for tau imaging. In this work, we have performed additional surface analysis for tau imaging to comprehensively evaluate and understand different AC methods. The surface map of the PET error image was generated for each tau-imaging dataset and was registered to the FSAverage template in Freesurfer to construct the averaged surface map.

For both regional and surface analysis, the relative PET error was used and calculated as

(2) $$PET_{\mathrm{error}\;}=\frac{\left|PET_{\mathrm{pseudoCT}\;}-PET_{\mathrm{trueCT}\;}\right|}{\left|PET_{\mathrm{trueCT}\;}\right|},$$

where *PET_pseudoCT_* and *PET_trueCT_* refer to the reconstructed PET images with AC using the pseudo-CT images generated from different MR AC methods and the ground-truth CT image, respectively. For regional analysis, *PET_pseudoCT_* and *PET_trueCT_* indicates the mean values inside the specified ROI. For surface analysis, *PET_pseudoCT_* and *PET_trueCT_* indicates the values at the vertices. With respect to the global PET analysis, Bland-Altman plots were drawn for both amyloid and tau imaging regarding SUVR to understand PET-error distributions of different methods.

### Comparison methods

One popular method available in clinical PET/MR scanners is the Dixon image-based atlas method [45]. This method is denoted as Atlas-Dixon and is adopted for comparison in this work. For the deep learning-based approach, apart from the proposed CNN-mUTE method, we have tested two other cases with different MR images: MPRAGE and Dixon images were used as network input for additional comparison, which are denoted as CNN-MPRAGE and CNN-Dixon, respectively.

## RESULTS

### Pseudo-CT analysis

**Fig. 1.**
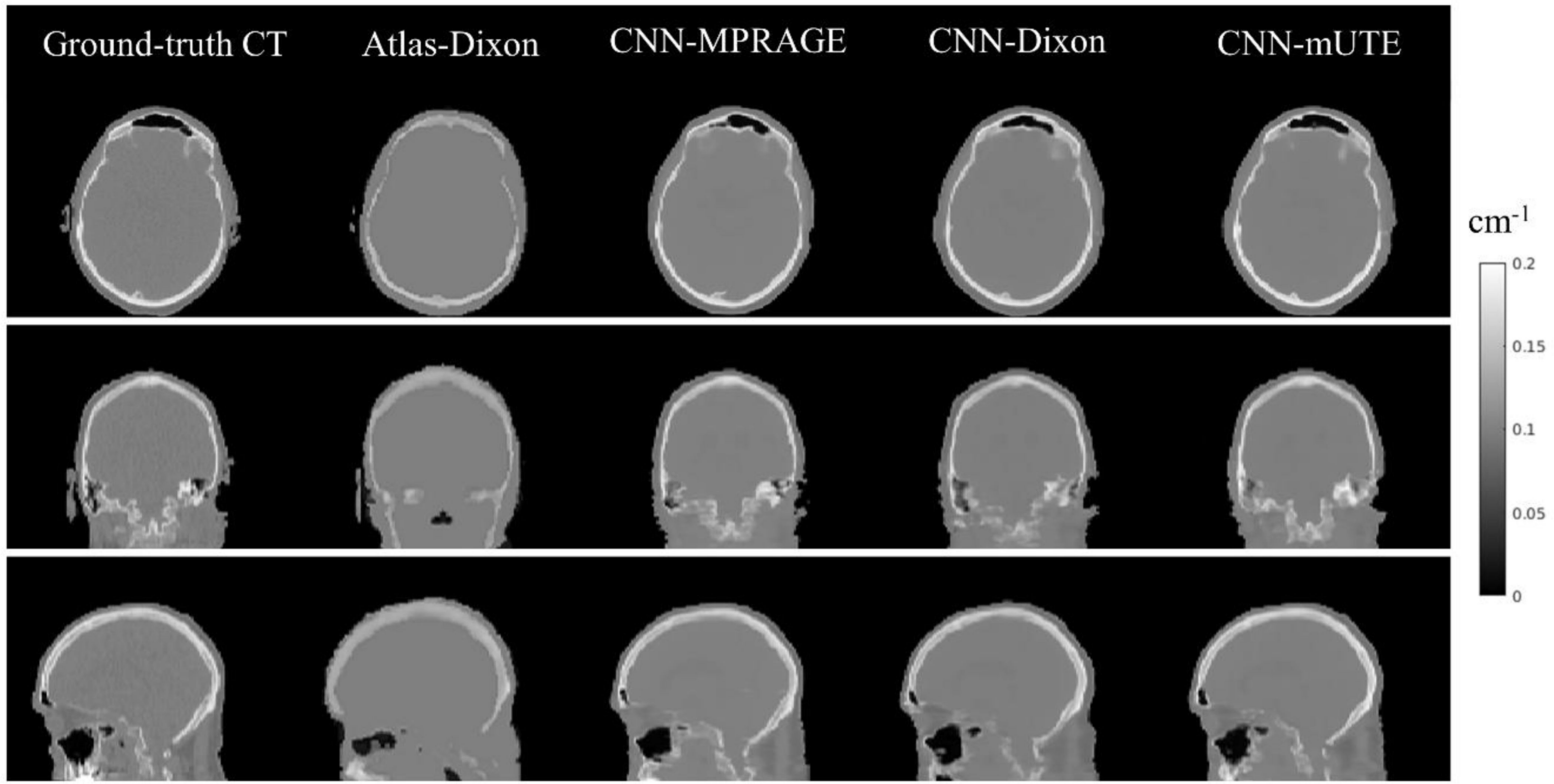
Three views of the ground-truth CT and the generated pseudo-CT images of one subject using different methods: Ground-truth CT (first column), Atlas-Dixon (second column), CNN-MPRAGE (third column), CNN-Dixon (fourth column) and CNN-mUTE (fifth column).

Fig. 1 shows one example of the generated pseudo-CT images using different methods along with the ground-truth CT image. All deep learning methods generated more accurate bone distributions compared to the Atlas-Dixon method, with CNN-mUTE revealing the most accurate bone details. For all thirty-five datasets, the relative validation loss and the Dice coefficients of the bone regions are shown in Table 1. As observed from the table, deep learning methods showed higher quantification accuracy compared to the Atlas-Dixon method. CNN-MPRAGE and CNN-Dixon showed similar performance and CNN-mUTE showed higher quantification accuracy compared to both CNN-MPRAGE and CNN-Dixon, regarding both relative validation loss and Dice coefficients.

### Reginal and global PET analysis

**Fig. 2.**
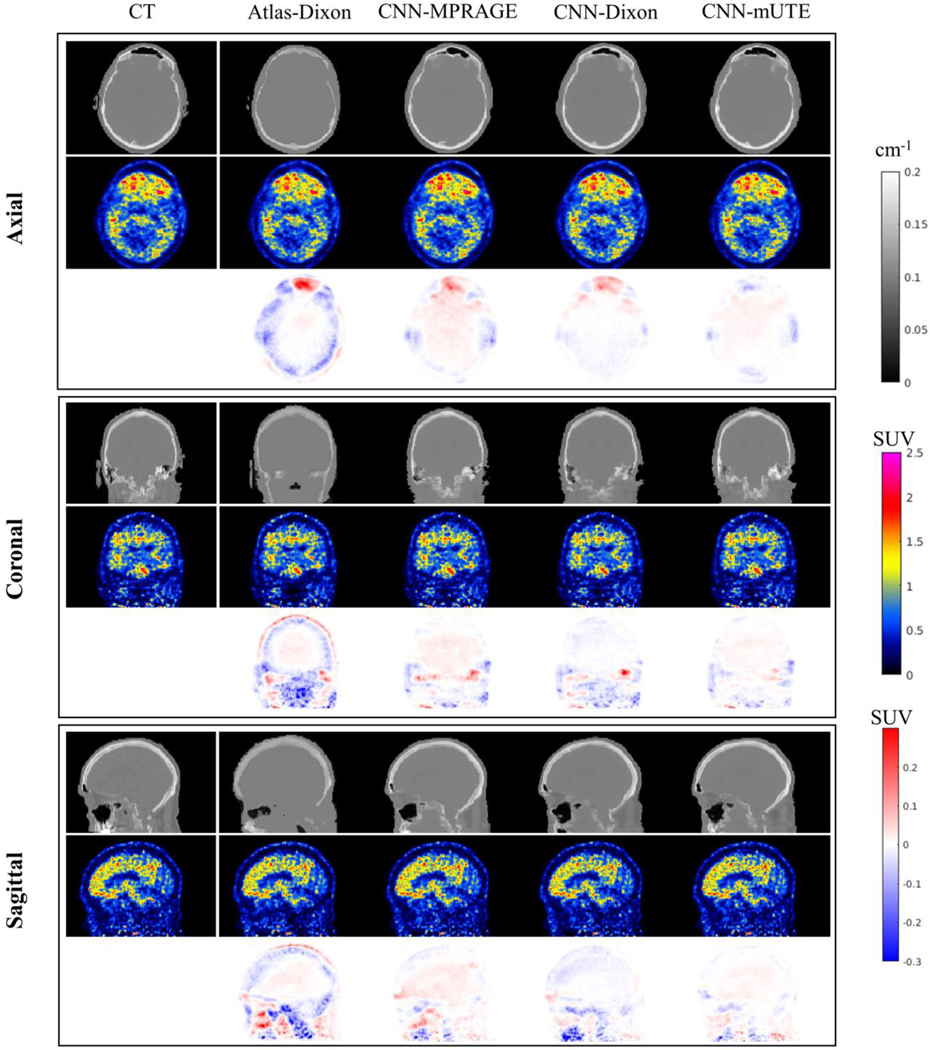
Three views of the CT/pseudo-CT images, PET images (^11^C-PiB, unit: SUV) and the corresponding PET error images (*PET_pseudoCT_* − *PET_trueCT_*, unit: SUV) for one subject. For each view, the first row shows the ground-truth CT image (first column) and the pseudo-CT images generated by Atlas-Dixon (second column), CNN-MPRAGE (third column), CNN-Dixon (fourth column) and CNN-mUTE (fifth column), respectively; the second row shows the PET images reconstructed using the ground-truth CT image and different pseudo-CT images; the third row shows the corresponding PET error images for different methods.

We have embedded the generated pseudo-CT images into PET image reconstruction to evaluate PET AC accuracy. Fig. 2 shows one amyloid-imaging example of the PET errors (*PET_pseudoCT_* − *PET_trueCT_*, unit: SUV) using different methods of the same subject as shown in Fig. 1. The CT/pseudo-CT images and the reconstructed PET images were also presented in the figure to better observe the source of errors. PET error images from the datasets with the largest and the smallest PET regional relative errors using the proposed CNN-mUTE method are shown in Supplementary Fig. 3 and Supplementary Fig. 4, respectively. These correspond to the best and worst cases using the proposed CNN-mUTE method. From these three examples, we can observe that the PET errors from the deep learning methods were smaller than those from the Atlas-Dixon method, especially alongside the bone regions.

**Fig. 3.**
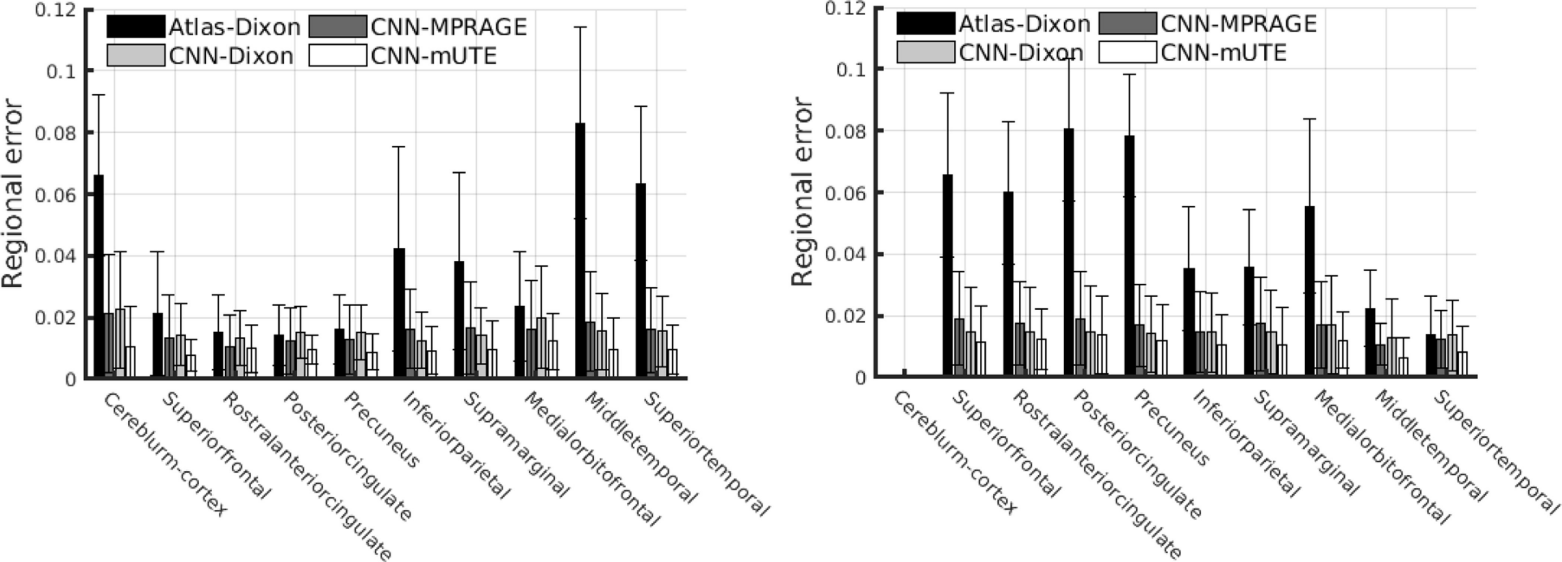
Regional analysis for amyloid imaging based on the ^11^C-PiB dataset for SUV (left) and SUVR (right).

**Fig. 4.**
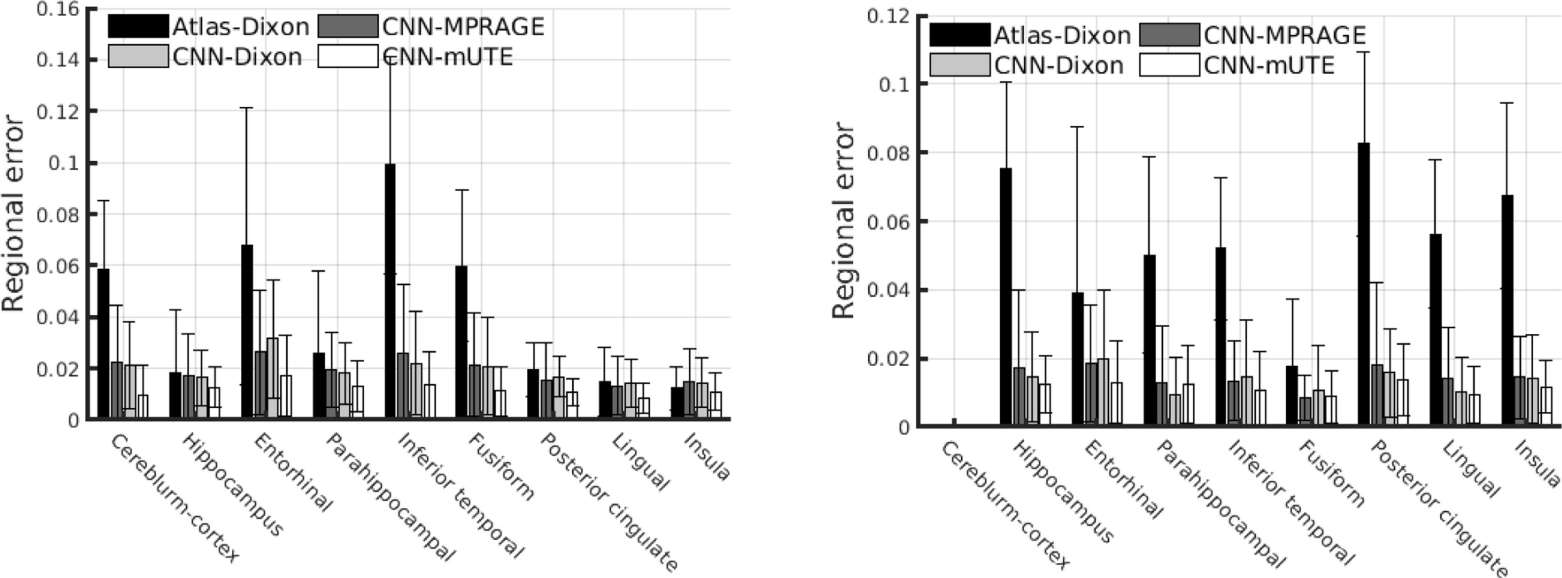
Regional analysis for tau imaging based on the ^18^F-MK6240 dataset for SUV (left) and SUVR (right).

Fig. 3 shows the regional PET analysis based on all thirty-five subjects from the amyloid-imaging dataset regarding SUV and SUVR. Fig. 4 shows the same analysis based on the tau-imaging dataset. The regional errors from all deep-learning methods were smaller than 2% for both amyloid and tau imaging. Although the SUV error in some cortex regions, e.g. hippocampus and posterior cingulate, were relatively small (e.g., lower than 2%) for the Atlas-Dixon method, those small-SUV-error cortex regions displayed much larger error for SUVR since the error in the cerebellum was significant (e.g., around 6%). As SUVR is the metric widely used for amyloid and tau imaging, the Atlas-Dixon method may cause significant errors for amyloid and tau imaging quantification.

**Fig. 5.**
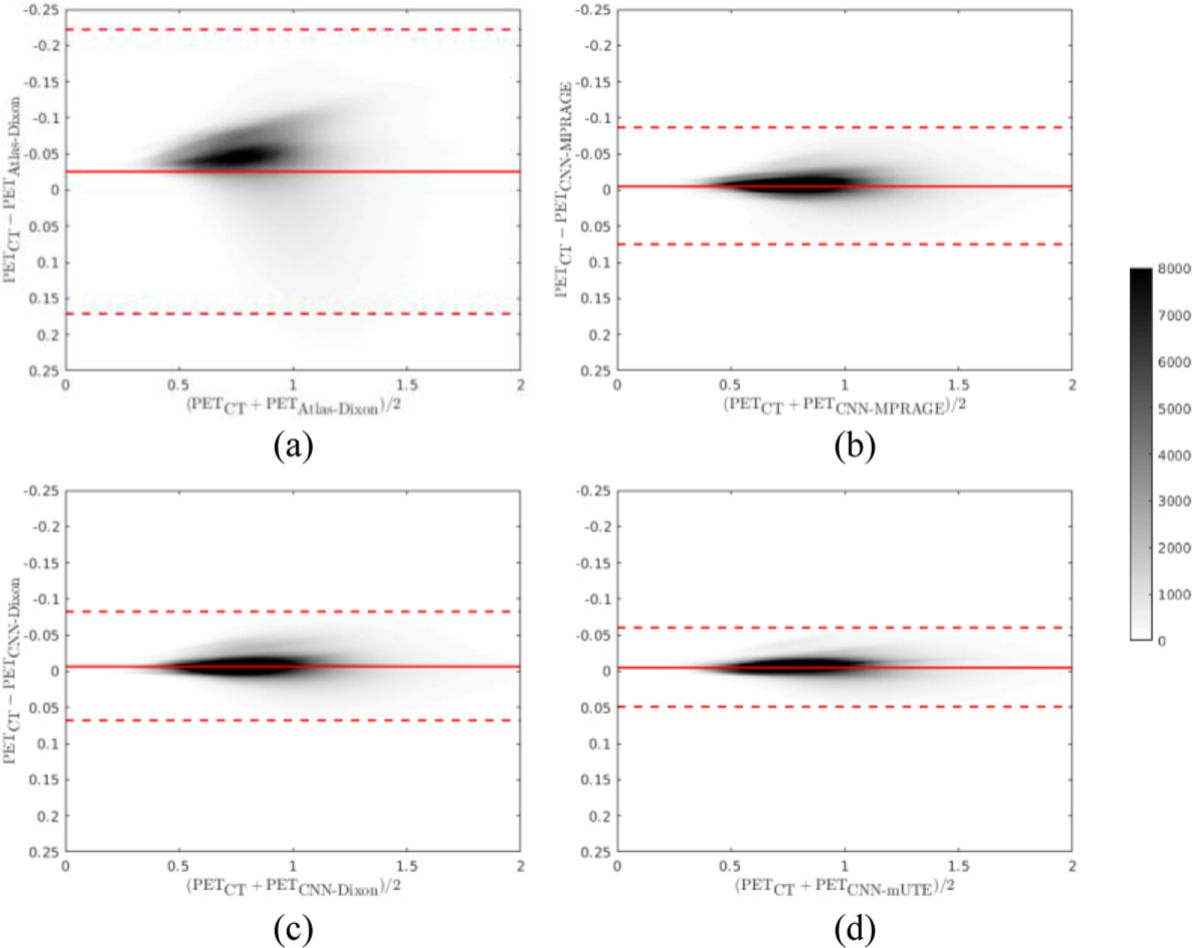
Bland-Altman plots regarding tau imaging for (a) Atlas-Dixon, (b) CNN-MPRAGE, (c) CNN-Dixon and (d) CNN-mUTE methods. The x-axis stands for the mean value between the PET images reconstructed using the ground-truth CT and generated pseudo-CT (0.5 ∗ (*PET_pseudoCT_* + *PET_trueCT_*), unit:SUVR). The y-axis stands for the difference between the PET images reconstructed using generated pseudo-CT and the ground-truth CT (*PET_pseudoCT_* − *PET_trueCT_*, unit: SUVR).

Fig. 5 shows the Bland-Altman plots for tau imaging regarding SUVR. Based on the distributions, we can see that the deep learning-based methods showed narrower error ranges compared with the Atlas-Dixon method. The error ranges from CNN-MPRAGE and CNN-Dixon were similar, and CNN-mUTE method showed relatively smaller error range compared to both CNN-MPRAGE and CNN-Dixon. The same trend was also observed from the Bland-Altman plot for amyloid imaging regarding SUVR presented in Supplementary Fig. 5.

### PET surface analysis

**Fig. 6.**
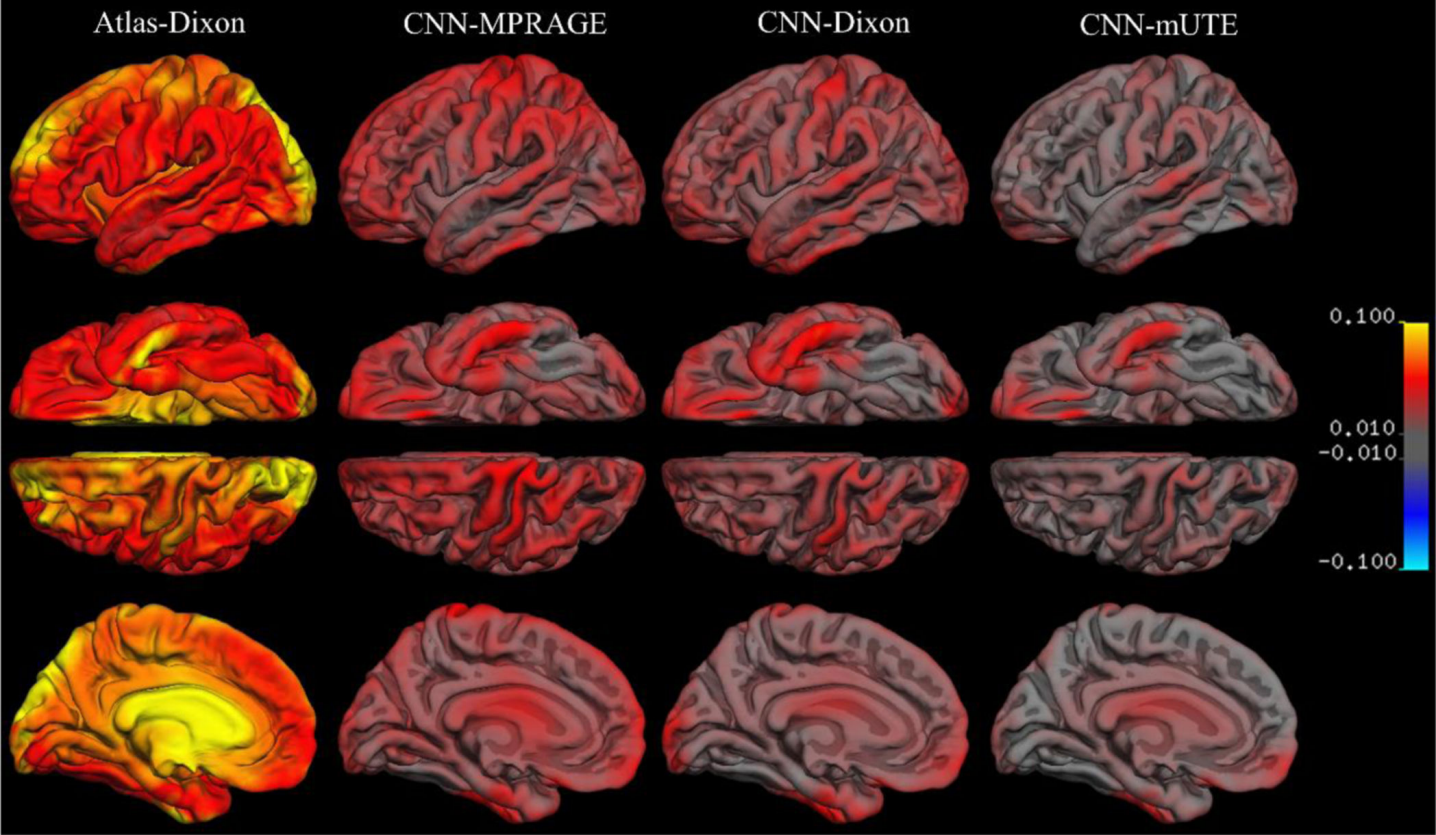
The averaged surface maps of SUVR relative error for different methods: Atlas-Dixon (first column), CNN-MPRAGE (second column), CNN-Dixon (third column) and CNN-mUTE (fourth column). Only the right hemisphere of the surface map is shown. The color map range is from 1% to 10% in magnitude.

Fig. 6 shows the averaged surface map for PET SUVR error of different AC methods for tau imaging. The Atlas-Dixon method showed the largest error (e.g., >10%) at vertices inside superior frontal, lateral occipital, superior parietal and inferior temporal cortices. The deep learning methods showed smaller error compared with the Atlas-Dixon method for almost all regions. For the deep learning methods, relatively high error (e.g., >5% ) was shown in some vertices near medial orbitofrontal and inferior temporal cortices. CNN-mUTE showed the least number of vertices with errors larger than 1%, followed by CNN-Dixon and CNN-MPRAGE.

## DISCUSSION

Compared to other PET applications, amyloid and tau imaging rely more on SUVR-based cortical quantification, which is significantly impacted by the accuracy of pseudo-CT bone map and the PET quantification in the cerebellar cortex. For tau imaging, in addition to regional analysis, evaluation of pixel-wise tracer distribution as well as the topographic patterns are necessary for better disease monitoring and early diagnosis. In this regard, specific evaluations of deep learning-based AC methods for amyloid and tau imaging are necessary. In this work, we have performed AC for amyloid and tau imaging using the proposed CNN-mUTE method, and compared it with deep learning methods based on Dixon and MPRAGE as well as the vendor-available Atlas-Dixon method through detailed regional and surface analysis. Results showed that for SUVR quantification, the Atlas-Dixon method showed relatively large errors. The deep-learning methods resulted in lower quantification error compared to the Atlas-Dixon method in both regional and surface analyses. Specifically, the proposed CNN-mUTE method provided better PET AC, in comparison to CNN-MPRAGE and CNN-Dixon methods. This is likely due to the abundance in physical information contained in the multi-contrast images from mUTE, which consist of perfectly registered UTE images for bone imaging and multi-echo Dixon images for robust water/fat separation. Our previous work has shown that a physical model can be derived to map the mUTE images to continuous attenuation coefficient distributions of bone, fat and water [22]. However, obtaining the model parameters requires solving a nonlinear estimation problem at each voxel, which becomes challenging in the presence of severe B0 and B1 inhomogeneities. Combining the mUTE-based AC method with deep learning further improves the accuracy and robustness of AC in PET/MR. All these results indicate that deep learning-based AC is a powerful technique to predict the attenuation map from MR images for amyloid and tau imaging, which can be further improved when network input is fed with more information of direct physical meaning.

The Group-Unet utilized in this work was developed to efficiently utilize multiple network inputs based on supervised learning [28]. We also developed a 3D Cycle-GAN network which did not need paired CT-MR images and can thus relax the training-data collection requirements [46]. Results presented in [46] showed that 3D Cycle-GAN was better than Dixon-based segmentation and atlas methods, and its performance was a little inferior to Group-Unet (Dice coefficient of the bone region was 0.76 for Group-Unet and 0.74 for Cycle-GAN). Because Cycle-GAN does not utilize the strong pixel-to-pixel loss from paired data, the results of Cycle-GAN are still very promising, especially for applications where registration or obtaining paired data is difficult. For this work, as paired CT-MR images were available, Group-Unet was preferred because of its better performance. In addition, the mUTE sequence generated multiple MR images. If Cycle-GAN was used, one of the generator networks need to synthesize multiple pseudo-MR images from one CT image, which might not be easy to train as it is a one-to-many mapping. Based on the consideration of network performance and training difficulty, Group-Unet was employed in this work.

As shown by Fig. 3 and Fig. 4, the cerebellar cortex can be largely influenced by the inaccuracy of bone in the pseudo-CT map,. This can be a problem especially for amyloid and tau imaging as SUVR, instead of SUV, is widely employed in amyloid and tau imaging to approximate the distribution volume ratio (DVR). The SUVR thresholds are often adopted to separate subjects into different clinical groups in amyloid and tau imaging. We noticed that the PET error maps for SUV and SUVR are quite different when Atlas-Dixon method was used for AC. Initially, we hypothesized that SUVR error may be smaller than SUV error as the errors in the cortex regions and the cerebellar cortex may cancel out. Results in this work demonstrated that this is not the case, as the error distribution for the cerebellar cortex and other cortices can be quite different. Deep learning-based AC methods can generate better bone maps near the cerebellar cortex and can greatly improve the overall SUVR quantification.

For the surface maps of tau imaging shown in Fig. 6, we noticed that for the medial orbitofrontal cortex and the entorhinal cortex, the deep learning-based methods still showed relatively large errors (e.g., >5%), though smaller than those from the Atlas-Dixon method. We presume this is because the bone near the sinuses and the mastoid part of the temporal bone are much more complicated than the cranial bone, which could be more difficult to be accurately synthesized through MR images. As medial orbitofrontal cortex is one of the composite cortex regions for amyloid burden calculation [44], and inferior and middle temporal cortices are crucial for early-stage tau quantification [42], further improvements of MR AC are still needed for amyloid and tau imaging. In addition, due to limited datasets, validation datasets were not used during network training. The epoch number was fixed at 600 for all network training. This epoch-number choice is not optimal, which is one limitation of current study.

## CONCLUSION

In this work, we have proposed an mUTE-based deep learning method for PET AC of amyloid and tau imaging. The performance of the proposed method was evaluated through regional and surface analyses on ^11^C-PiB and ^18^F-MK6240 datasets acquired on thirty-five subjects. Our results show that deep learning-based methods can generate accurate AC for SUVR quantification, with the proposed mUTE-based deep learning method achieving the best AC performance when compared to MPRAGE- and Dixon-based deep learning methods as well as the Dixon-based atlas method.

## Supplementary Material

259_2020_5061_MOESM1_ESM

## Figures and Tables

**Table 1.** Quantification of the generated pseudo-CT, regarding the validation loss and Dice coefficient of the bone regions.

Methods	Relative validation loss (%)	Dice of bone whole	Dice of bone above eye	Dice of bone below eye
**Atlas-Dixon**	28.72±0.02	0.52±0.06	0.64±0.08	0.19±0.07
**CNN-MPRAGE**	12.58±0.01	0.84±0.03	0.91±0.02	0.70±0.07
**CNN-Dixon**	11.44±0.01	0.84±0.03	0.92±0.02	0.68±0.06
**CNN-mUTE**	**10.94**±0.01	**0.87**±0.03	**0.94**±0.02	**0.73**±0.06
