# Supplementary material for: Attenuation Correction Using Deep Learning and Integrated UTE/Multi-Echo Dixon Sequence: Evaluation in Amyloid and Tau PET Imaging
Source: Eur J Nucl Med Mol Imaging. Author manuscript; Available in PMC 2022 May 1. (PMC8411350; doi:10.1007/s00259-020-05061-w)
Supplement: 259_2020_5061_MOESM1_ESM [file NIHMS1641682-supplement-259_2020_5061_MOESM1_ESM.docx]

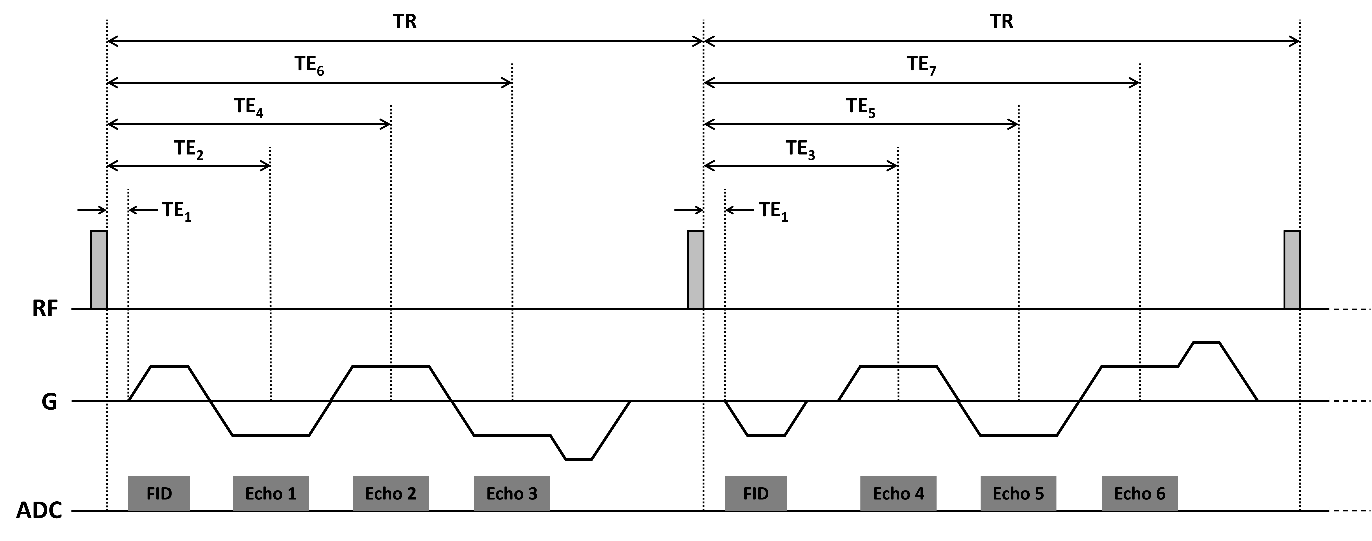


Supplementary Fig.1. Schematic diagram of the mUTE sequence. The sequence consists of UTE and alternating acquisition of multi-echo Dixon images every other TR along radial k-space trajectories. The shown example results in seven images: one UTE image and six multi-echo Dixon images at different TEs.

Supplementary Fig. 2. The Group-Unet structure used in this work: (a) the network structure; (b) the group convolutional module used in the network structure shown in (a); (c) the explanation of numbers in each of the block in the group convolution module shown in (b) .


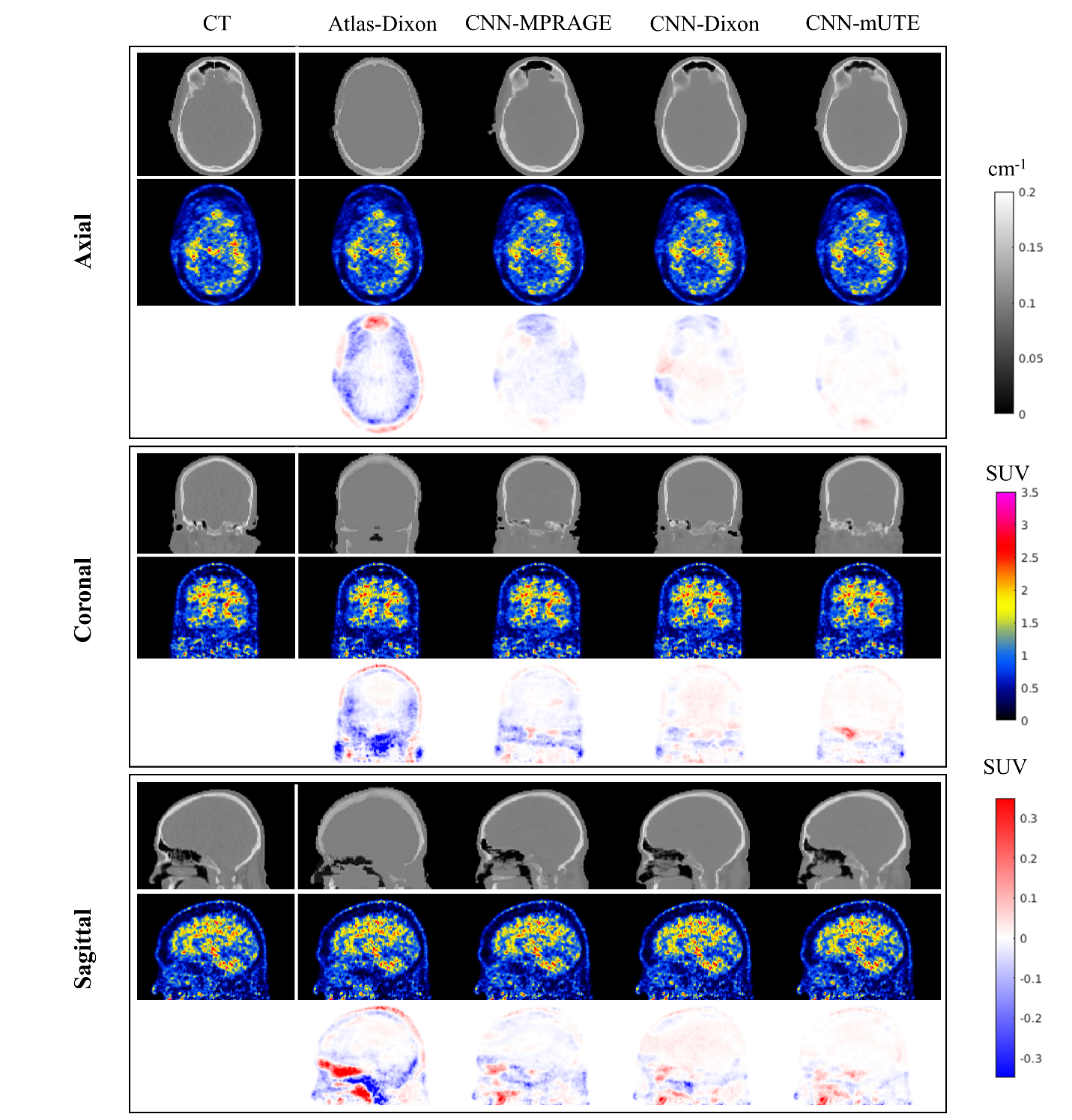


Supplementary Fig. 3. Three views of the CT/pseudo-CT images, PET images (^11^C-PiB, unit: SUV) and the corresponding PET error images (${PET}_{pseudoCT}-{PET}_{trueCT}$, unit: SUV) for one subject, whose total PET relative errors (summation of the relative errors in superior frontal, rostral anterior cingulate, posterior cingulate, precuneus, inferior parietal, supramarginal, medial orbitofrontal, middle temporal and superior temporal) using the CNN-mUTE method is the smallest. For each view, the first row shows the ground-truth CT image (first column) and the pseudo-CT images generated by Atlas-Dixon (second column), CNN-MPRAGE (third column), CNN-Dixon (fourth column) and CNN-mUTE (fifth column), respectively; the second row shows the PET images reconstructed using the ground-truth CT image and different pseudo-CT images; the third row shows the corresponding PET error images for different methods.


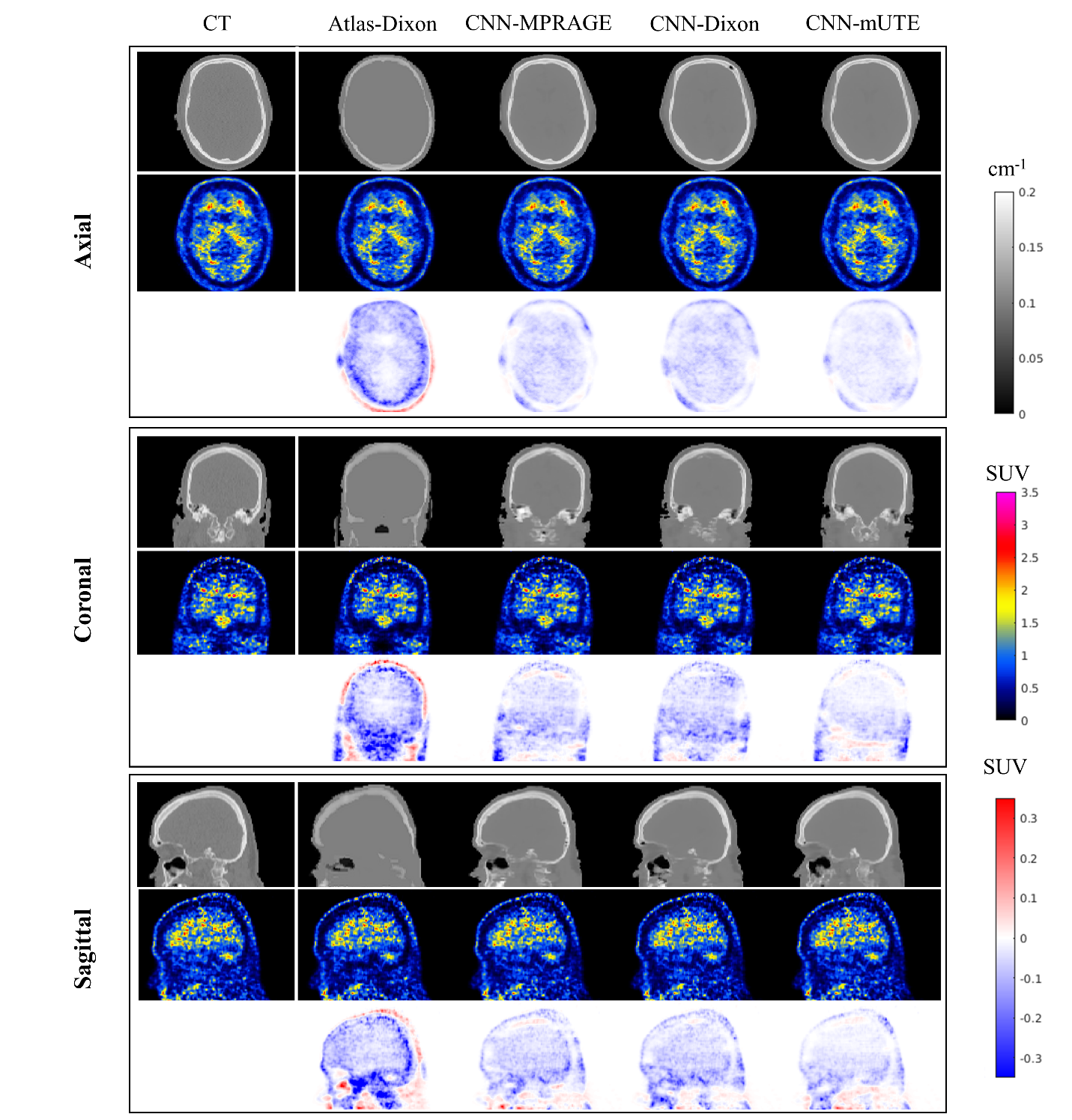


Supplementary Fig. 4. Three views of the CT/pseudo-CT images, PET images (^11^C-PiB, unit: SUV) and the corresponding PET error images (${PET}_{pseudoCT}-{PET}_{trueCT}$, unit: SUV) from one dataset, whose total PET relative errors (summation of the relative errors in superior frontal, rostral anterior cingulate, posterior cingulate, precuneus, inferior parietal, supramarginal, medial orbitofrontal, middle temporal and superior temporal) using the CNN-mUTE method is the largest. For each view, the first row shows the ground-truth CT image (first column) and the pseudo-CT images generated by Atlas-Dixon (second column), CNN-MPRAGE (third column), CNN-Dixon (fourth column) and CNN-mUTE (fifth column), respectively; the second row shows the PET images reconstructed using the ground-truth CT image and different pseudo-CT images; the third row shows the corresponding PET error images for different methods.


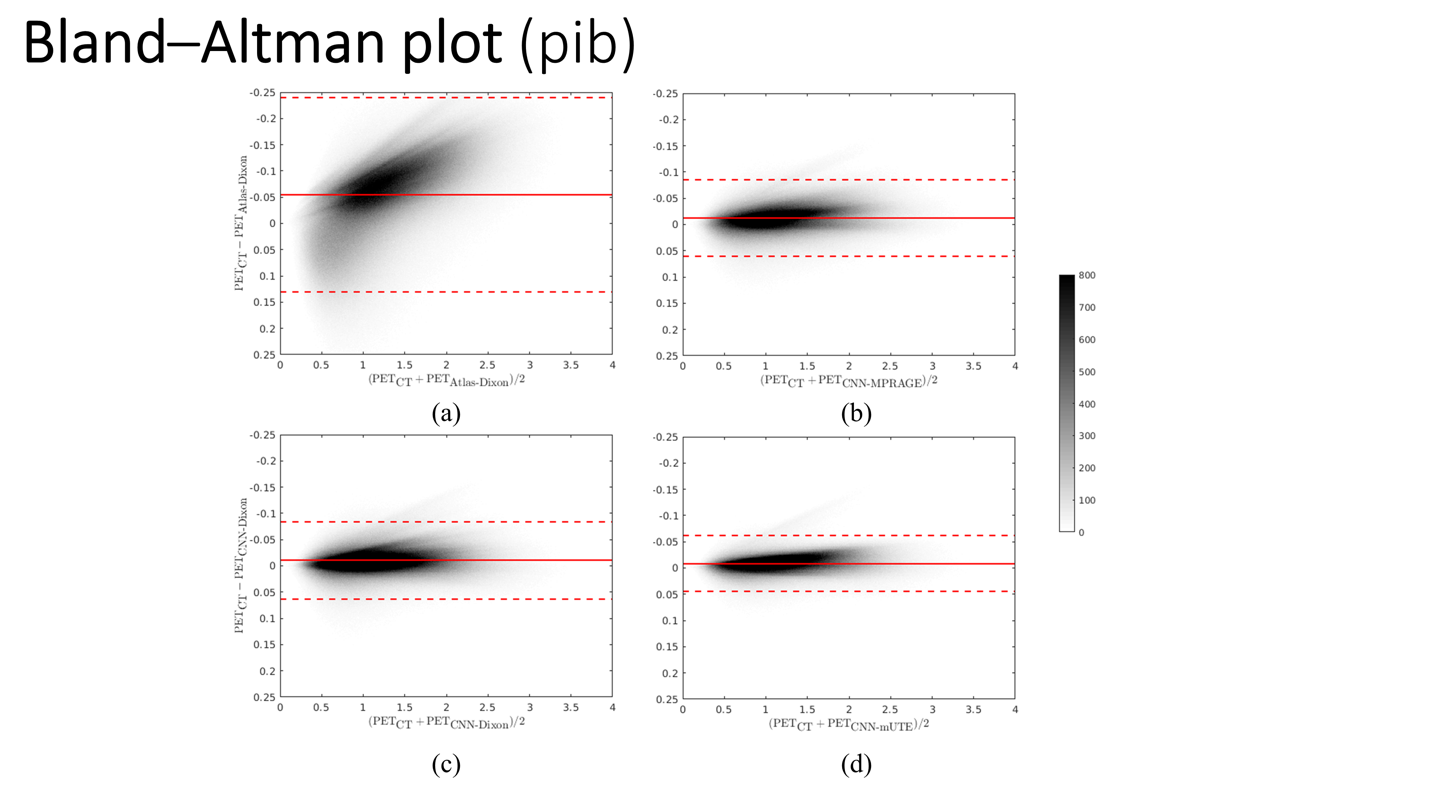


Supplementary Fig. 5. The Bland-Altman plots regarding amyloid imaging for (a) Atlas-Dixon, (b) CNN-MPRAGE, (c) CNN-Dixon and (d) CNN-mUTE methods. The x-axis stands for the mean value between PET images reconstructed using the ground-truth CT and generated pseudo-CT (${0.5*(PET}_{pseudoCT}+{PET}_{trueCT})$, unit: SUVR). The y-axis stands for the difference between PET images reconstructed using the ground-truth CT and generated pseudo-CT (${PET}_{pseudoCT}-{PET}_{trueCT}$, unit: SUVR).
